# Adaptive Boosting Based Personalized Glucose Monitoring System (PGMS) for Non-Invasive Blood Glucose Prediction with Improved Accuracy

**DOI:** 10.3390/diagnostics10050285

**Published:** 2020-05-07

**Authors:** Pradeep Kumar Anand, Dong Ryeol Shin, Mudasar Latif Memon

**Affiliations:** 1 College of Information and Communication Engineering, Sungkyunkwan University, Suwon 16419, Korea; pradeep@skku.edu; 2 College of Software, Sungkyunkwan University, Suwon 16419, Korea; 3 IBA Community College Naushahro Feroze, Sukkur IBA University, Sindh 65200, Pakistan; mudasarlatif.ccnf@iba-suk.edu.pk

**Keywords:** diabetic care, non-invasive blood glucose monitoring, personalized calibration, machine learning, adaptive boosting, clustering, error prediction model

## Abstract

In this paper, we present an architecture of a personalized glucose monitoring system (PGMS). PGMS consists of both invasive and non-invasive sensors on a single device. Initially, blood glucose is measured invasively and non-invasively, to train the machine learning models. Then, paired data and corresponding errors are divided scientifically into six different clusters based on blood glucose ranges as per the patient’s diabetic conditions. Each cluster is trained to build the unique error prediction model using an adaptive boosting (AdaBoost) algorithm. Later, these error prediction models undergo personalized calibration based on the patient’s characteristics. Once, the errors in predicted non-invasive values are within the acceptable error range, the device gets personalized for a patient to measure the blood glucose non-invasively. We verify PGMS on two different datasets. Performance analysis shows that the mean absolute relative difference (MARD) is reduced exceptionally to 7.3% and 7.1% for predicted values as compared to 25.4% and 18.4% for measured non-invasive glucose values. The Clarke error grid analysis (CEGA) plot for non-invasive predicted values shows 97% data in Zone A and 3% data in Zone B for dataset 1. Moreover, for dataset 2 results echoed with 98% and 2% in Zones A and B, respectively.

## 1. Introduction

As per the World Health Organization (WHO), approximately 422 million people have diabetes, and around 1.6 million people died from it in the year 2014 [1]. The number of diabetic patients is growing every year at an approximate rate of 8.5%. At this rate, it is expected that by 2030 more than a billion people will have diabetes mellitus. WHO has declared diabetes as the number one disease in the world [1].

Diabetes is caused by insulin disorder. The causes of insulin disorder can be by birth or due to an unhealthy diet and low physical activity. These two causes are primarily responsible for Type 1 and Type 2 diabetes, respectively [2]. Type 1 diabetes (also referred to Juvenile-onset) is caused due to ineffective production of insulin produced by beta cells of the pancreas whereas Type 2 diabetes (also known as adult-onset) arises due to the ineffective use of insulin inside the body. The main reason for Type-2 diabetes is a metabolic disorder due to high blood glucose involving insulin resistance. According to WHO, Type-2 diabetes is responsible for approximately 90% of all diabetes cases [1,2]. Both Type-1 and Type-2 diabetic patients need a diagnosis and regular monitoring to manage their disease by measuring blood glucose levels. Different biological mediums can be used to measure blood glucose levels inside the human body. These mediums are saliva, tears, sweat, urine, and blood [3,4,5,6,7]. The highest glucose concentration is present in the blood; hence, it is the best biological medium for blood glucose measurement [7].

To date, several invasive monitors are developed, which are highly accurate and become a gold standard to measure blood glucose [8]. Some of these monitors are Contour Next, Accu-check Aviva plus, Freestyle Lite. These monitors have >95% accuracy and hence meet the requirement as defined by the Food and Drug Administration (FDA) [9]. However, the invasive monitor requires to prick the finger for every measurement. This method is a painful process and leaves several scars on the body of a patient. Therefore, a real need exists for an accurate non-invasive monitoring system. To satisfy this need, different non-invasive sensors were developed in the last few decades. Some of the non-invasive sensing technologies used for blood glucose measurements are infrared [10], impedance [11], diffuse reflectance [12], Raman spectroscopy [13], optical coherence tomography [14], photo-acoustic spectroscopy [15], and combination of sensors [16,17,18]. Using these sensors, different non-invasive or minimally invasive monitors are developed. At present, non-invasive or minimally invasive monitors are GlucoTrack [18], Combo Glucometer [19], SugerBEAT [20], Symphony [21], Wizmi [22], Eversense [23]. Even though tremendous development occurred in non-invasive sensing technology, unfortunately, these non-invasive monitors do not meet the >95% accuracy requirement defined by the FDA [24,25,26,27,28].

On the other hand, machine learning becomes powerful due to the presence of high computational capabilities. One of the goals of machine learning is to predict the data, as accurately as possible by creating a model based on existing data after extracting relevant features [29,30]. Regression is a very common and important task in machine learning to predict using the trained model. There are many different kinds of machine learning regression algorithms, which can be used for prediction. Some of these methods are linear regression, support vector machine (SVM), K-nearest neighbors, decision tree, random forest, and adaptive boosting [31,32]. Linear regression, support vector machine, k-nearest neighbors, and decision tree are widely used to solve simple regression problems [31,32]. However, ensemble methods like random forest and AdaBoost are used to combine the prediction of multiple machine learning models that are individually weak to produce a more accurate forecast [33,34,35,36,37].

Therefore, we develop and present the concept of the personalized glucose monitoring system (PGMS) using machine learning for the accurate measurement of blood glucose by the non-invasive method. To be more specific, the following are our contributions:

(1) We develop an architecture, working principle, and software for PGMS. It consists of invasive and non-invasive sensors on a single device;
(2) PGMS software forms different clusters consisting of invasive and non-invasive measured paired data. These clusters are developed scientifically based on domain-knowledge as per blood glucose ranges mapped with diabetes patient condition named as “hypoglycemia, normal, pre-diabetic, diabetic, highly diabetic, and critically diabetic”;
(3) We develop the error prediction model for each cluster using the AdaBoost algorithm to predict the non-invasive blood glucose value accurately with goals to achieve the least overall MARD and Root Mean Square Error (RMSE).

We make the trained and optimized AdaBoost models to undergo personalized calibration for any patient based on his/her characteristics. After calibration, a patient can measure his/her blood glucose level by the non-invasive method only. Our proposal reduces the MARD of less than 10% on two different datasets. We also present the results graphically by using the CEGA plot, the graphs delineate that our proposed PGMS has more than 95% of the data in Zone A; less than 5% in Zone B; and none in Zones C, D, and E for both datasets.

The rest of the paper is organized as follows. Section 2 details the related work by describing the tools for blood glucose accuracy assessment, accuracy regulation around the world, existing invasive and non-invasive blood glucose monitoring systems, and details of the AdaBoost algorithm. Section 3 consists of the proposed architecture, working principle, development of error prediction model using the AdaBoost algorithm, software implementation, and baseline simulation data details. Section 4 consists of initial approaches with challenges, the final concept of the PGMS, test results, and discussion. Finally, Section 5 concludes the paper.

## 2. Related Work

In this section, we present the summary of prior works done in the area of blood glucose measurement and the AdaBoost machine learning model, which is related to our proposed work on the PGMS.

### 2.1. Parameters to Assess the Accuracy of Non-Invasive Blood Glucose Monitoring System

#### 2.1.1. Mean Absolute Relative Difference (MARD)

The absolute percentage of errors can be calculated for all non-invasive values with respect to reference invasive blood glucose values. The average value of all absolute percentage errors results in the MARD as calculated by Equation (1). The lower the MARD, the higher the device’s accuracy. It is a simple method to assess the accuracy of the non-invasive blood glucose measuring device, but it highly depends on the number of paired data points. Some of the highly inaccurate non-invasive values may not be visible due to the average calculation on a large data set. Hence, MARD cannot be used as the sole assessment parameter to determine the accuracy of the device [38,39].

(1) $$MARD=\frac{100\%}{N}\times \sum_{k=1}^{N}\frac{\left|y_{ni-k}-y_{ref-k}\right|}{y_{ref-k}}$$

Here,

$y_{ni-k}$ = Non-invasive blood glucose value at k^th^ measurement
$y_{ref-k}$ = Reference invasive blood glucose value at k^th^ measurement
k = Measurement number, which is 1,2….N
N = Total number of measurements
MARD = Mean absolute relative difference

#### 2.1.2. Clarke Error Grid Analysis (CEGA) Plot

**Figure 1:**
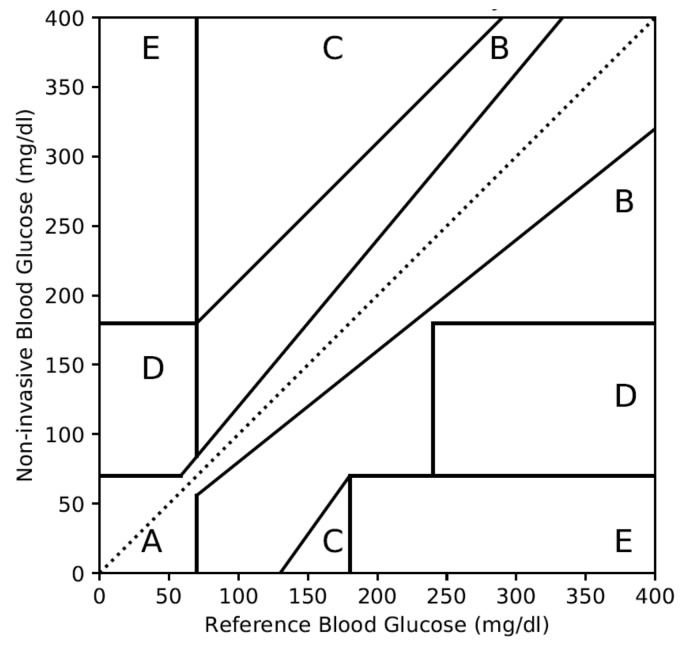
Clarke error grid analysis (CEGA) plot with different zones.

In 1987, Dr. William L. Clarke established the method for the assessment of the accuracy of self-monitoring blood glucose devices [40]. As per his method, each of the non-invasive blood glucose values should be plotted to respective reference values, as shown in Figure 1. Then based on the clinical criticality of the hypoglycemia and hyperglycemia conditions, the plot is divided into five zones. These five zones are explained below:

- Zone A: Deviation with respect to reference is <20%. Values falling within this range are clinically accurate with the right treatment.
- Zone B: Deviation with respect to reference is >20% and within the range of clinically benign condition without risk to the patient.
- Zone C: Deviation is within hypoglycemia and hyperglycemia ranges. However, it can lead to unnecessary treatment.
- Zone D: Deviation is potentially dangerous and can fail to detect the hypoglycemic or hyperglycemic situation.
- Zone E: Deviation is extremely dangerous and can confuse hypoglycemia for hyperglycemia or vice versa.

#### 2.1.3. Minimum and Maximum Percentage Error

The minimum and maximum relative differences are the possible extreme errors by a non-invasive blood glucose monitor. These parameters are also important to assess the accuracy of a non-invasive blood glucose monitoring device.

### 2.2. Regulation for Blood Glucose Measurement Accuracy

Blood glucose measurement and accuracy regulations around the world are very stringent to prevent the case of hypoglycemia and hyperglycemia. Table 1 shows different regulations around the world and expected device accuracy to measure blood glucose.

These regulations apply to both invasive and non-invasive blood glucose monitoring devices. As per the US FDA, if the error in blood glucose measurement ranges from −15% to 15%, then overall device accuracy must be greater than 95%, and MARD should be less than 5% [24,25]. However, if the measurement error is in the range of −20% to 20%, then overall device accuracy must be more than 99%. Similarly, the European Medicines, Health Canada, and China FDA regulation define more than 95% of device accuracy and error ranges from −15% to 15% for blood glucose values ≥100 mg/dL [26,27,28]. In case the blood glucose value is <100 mg/dl, then the acceptable error range is −15 to 15 mg/dl with device accuracy greater than 95% and MARD less than 5%.

### 2.3. Accuracy of Invasive Blood Glucose Monitoring Devices

In the last few decades, several invasive blood glucose self-monitoring devices were developed that meet FDA accuracy requirements as defined in Table 1. Table 2 lists some of FDA approved, highly accurate, and commercially available invasive self-blood glucose monitors.

Contour Next from Bayer tops the list and is a highly accurate invasive blood glucose monitor with 100% accuracy as listed in Table 2 [9]. Accu-check Aviva plus is another popular invasive device from Roche with 99% accuracy [9]. These invasive devices are easy to use by a patient to measure his/her blood glucose value at home. However, a patient has to prick the skin every time to measure the blood glucose, and the skin of a diabetic patient takes more time to heal than normal.

### 2.4. Accuracy of Non-Invasive and Minimal-Invasive Blood Glucose Monitoring Devices

In this section, we present the accuracy study of existing non-invasive sensors and monitoring devices.

#### 2.4.1. Accuracy of Non-Invasive Blood Glucose Sensor Technologies

In the last few decades, several non-invasive sensors were developed using various technologies to measure blood glucose as shown in Table 3. Infrared and impedance spectroscopy were the first few non-invasive sensors, which were developed to measure blood glucose [10,11]. Accuracies of these two sensing technologies to measure the blood glucose are 84% and 56%, respectively, in Zone A of the CEGA plot as listed in Table 3. Zone A can have data points with error in non-invasive measurements up to ± 20% compared to the reference. It means, both infrared and impedance spectroscopy sensors have an extensive error, and hence, they cannot meet FDA regulation of ±15% error with 95% device accuracy.

Other sensing technologies like diffuse reflectance [12], Raman spectroscopy [13], optical coherence tomography [14], and photo-acoustic spectroscopy [15] achieved accuracies around 87.5%, 86.7%, 83%, and 82.7% in Zone A of CEGA plot, respectively. It means none of these sensors alone meets the FDA requirements. Even a combination of multiple non-invasive blood glucose sensors, like Multi-sensor1 [16], Multi-sensor2 [17], Multi-sensor3 [18] on a single measuring device does not meet the accuracy requirement as defined by the FDA. Though tremendous development in non-invasive sensing technologies and algorithms happened, it cannot meet the FDA and other countries’ regulations.

#### 2.4.2. Accuracy of Non-Invasive Blood Glucose Monitoring Devices

We investigated some of the self-monitoring non-invasive blood glucose devices as listed in Table 4. Integrity application GlucoTrack design achieves the accuracy for the non-invasive blood glucose measurement by combining the output of three different types of sensors based on ultrasonic, electromagnetic, and thermal technology mounted on a single device. However, GlucoTrack achieves MARD of 23.4% for non-invasive measurements [18] and hence does not meet FDA accuracy requirement of <5% of MARD.

Combo Glucometer (CoG) by Cnoga Medical is one of the best performing non-invasive blood glucose monitors commercially available. It uses near-infrared spectroscopy technology. It consists of four LEDs and four sensors to analyze absorption and scattering patterns. The sensor data is analyzed using a neural network algorithm. Even though it is one of the best performing devices, it has a MARD of 17.1% [19], which is still much higher than the FDA requirement.

SurgerBEAT, Symphony, Wizmi, and Eversense are minimally-invasive monitors. They have achieved MARD of 13.8%, 18.3%, 7.2%, and 14.8%, respectively [20,21,22,23]. All these monitors are under development for continuous blood glucose monitoring.

Hence, all of the above-mentioned devices do not meet the 95% accuracy or 5% MARD requirement as defined by the FDA as well as the regulatory organization of other countries.

### 2.5. Machine Learning Based AdaBoost Algorithm for Prediction

In 1990, Yoav Freund and Robert Schapire proposed and proved that the boosting algorithm is a method that uses pre-generated weak predictors for continuous learning, gradually boosting them as “strong predictors” [33]. They introduced the regression algorithm named as AdaBoost.R. Later, Harris Drucker modified AdaBoost.R regression technique and introduced a new technique named as AdaBoost.R2 to achieve higher prediction efficiency [34]. Hence, we use AdaBoost.R2 algorithm in our approach. The following steps are involved in AdaBoost.R2 algorithm:

- Input: The AdaBoost regressor starts with a weak predictor, such as a decision tree, based on an input dataset (u_i_, v_i_).
  Sequence of m examples (u_1_,v_1_),…, (u_m_,v_m_) where labels v_i_ ∈ R
  Weak learning algorithm Weak Learner
- Initialize: Next, the initialization in AdaBoost starts with equal weights for all datasets. We consider average loss function as zero during the first iteration.
  Iteration t = 1
  Distribution D_t_(i) = 1/m for all i
  Average loss function L_tavg_ = 0
- Iterate: Then, the weak predictor is called repeatedly, making the predictor more concerned with samples that are difficult to predict by giving greater weight to incorrectly predicted samples in each round. During this process, the average loss function is calculated by linear or square, or exponential law depends on data as shown in Equations (3)–(5).
  Average loss function L_tavg_ < 0.5
  Call f_t_(u_i_), providing it with distribution D_t_
  Build the regression model: f_t_(u_i_)⟶v_i_
  *Calculate the loss for each training example as,*
    (2) $$l_{t}(i)=\left|f_{t}(u_{i})-v_{i}\right|$$
  *Calculate the loss function L_t_(i) for each training example using any of 3 functional forms as,*
    (3) $$\mathrm{Linear}:\;L_{t}(i)=\frac{l_{t}(i)}{Den_{t}}$$
    (4) $$\mathrm{Square}:\;L_{t}(i)={\left(\frac{l_{t}(i)}{Den_{t}}\right)}^{2}$$
    (5) $$\mathrm{Exponential}:\;L_{t}(i)=1-e^{\left(\frac{-l_{t}(i)}{Den_{t}}\right)}$$
    (6) $$\mathrm{Where},\;Den_{t}(i)=\sup\left|f_{t}(u_{i})-v_{i}\right|$$
  *Calculate an average loss from Equation (7),*
    (7) $$L_{tavg}=\sum_{i=1}^{m}L_{t}(i)D_{t}(i)$$
  *Calculate the measure of confidence in predictor as per Equation (8),*
    (8) $$\beta_{t}=\frac{L_{tavg}}{1-L_{tavg}}$$
  *Update distribution D_t_ by using Equation (9),*
    (9) $$D_{t+1}(i)=D_{t}(i)\times \beta_{t}{}^{\left(1-L_{t}(i)\right)}$$
  *Set t = t + 1 to perform next iteration*.
- Output: For a particular input u_i_, each of the T machines makes a prediction f_t_. The final hypothesis is formed using T predictors as shown in Equation (10).
  (10) $$f_{final}(u)=\inf\left[v\in R:\sum_{t:f_{t}(u)\leq v}\log\left(\frac{1}{\beta_{t}}\right)\geq \frac{1}{2}\sum_{t}\log\left(\frac{1}{\beta_{t}}\right)\right]$$

Here,
$u_{i}$ = Independent variables for i^th^ data;$v_{i}$ = Dependent variables for i^th^ data;i = Number of data, which varies from is 1,2….m;m = Total number of data;t = Iteration number, which varies from 1,2….T;T = Total number of iterations;D_t_(i) = Probability distribution for i^th^ data at t^th^ iteration;f_t_(u_i_) = Weak predictor of variable u_i_ at t^th^ iteration;$l_{t}\left(i\right)$ = Loss of each training data at t^th^ iteration;L_tavg_ = Average loss function at t^th^ iteration;L_t_(i) = Loss function of each training data at t^th^ iteration;β_t_ = Measure of confidence in predictor at t^th^ iteration;$f_{final}$ = Strong or final predictor;


Motivated by the prediction accuracy of the AdaBoost algorithm on similar kinds of input data [33,34,35,36,37], we proceed to use the AdaBoost to build an error prediction model between non-invasive blood glucose values with respect to reference invasive values in our application. 

## 3. Machine Learning Based PGMS with Improved Accuracy

We present a concept of the machine learning based PGMS system that allows a patient to measure his blood glucose level non-invasively with more accuracy.

### 3.1. PGMS Architecture

**Figure 2:**
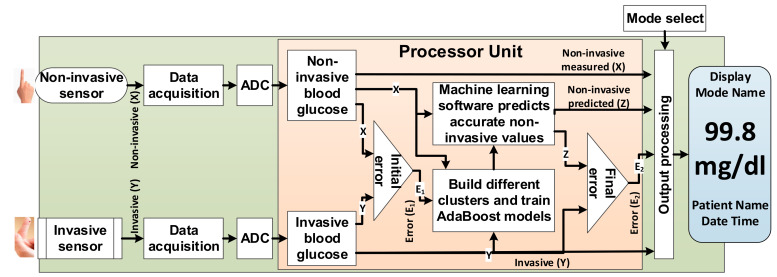
Personalized glucose monitoring system (PGMS) structural diagram.

We propose the architecture of PGMS, as shown in Figure 2. Our proposal has both invasive and non-invasive sensors on a single device to calculate the initial errors (E_1_) in non-invasive measured values (X) with respect to reference invasive values (Y) as shown in Figure 2. The processor unit divides the measured paired data into different clusters. Our approach then uses a Machine learning based AdaBoost algorithm to train the models based on non-invasive measured values (X) and reference values (Y) to predict non-invasive values (Z) for each cluster. The final error (E_2_) is calculated based on non-invasive predicted (Z) with respect to a reference value (Y), as shown in Figure 2. All these invasive, non-invasive measured, non-invasive predicted, initial error, and final error are shown on a display. A mode select button helps the user to choose invasive or non-invasive measurement methods. 

### 3.2. Working Principle of the PGMS

**Figure 3:**
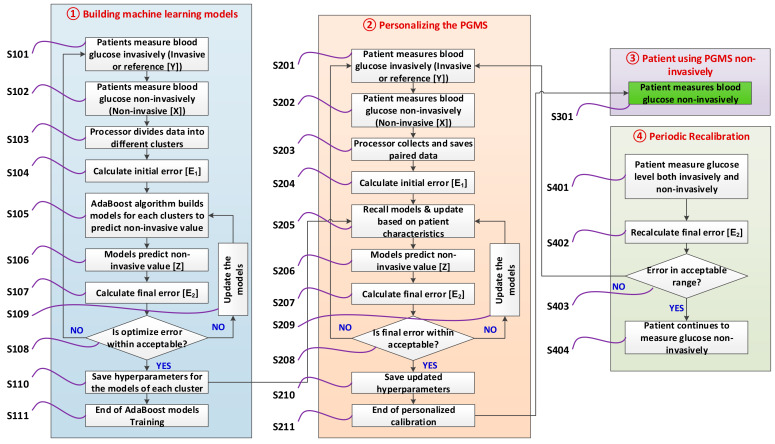
Software flowchart of the PGMS.

The working principle of the PGMS is explained in four stages, as shown in Figure 3. Let us discuss each stage in detail below.

#### 3.2.1. Building Machine Learning Models

Initially, PGMS builds different machine learning models. For each patient, we measure blood glucose, both non-invasively (X) and invasive (Y), as shown in S101 and S102 of Figure 3. In step S103, the processor collects and saves the paired data (X, Y) in different clusters. Initial error (E_1_) is calculated for each pair of data (X, Y), as shown in step S104. Several paired data are collected. We developed the machine learning software to build an error prediction model using initial errors in non-invasive measured values. The purpose of the software is to train the AdaBoost consisting of error prediction models for each cluster as described in step S105.

The output of the AdaBoost models is to predict accurate non-invasive blood glucose value (Z), as demonstrated in step S106 of Figure 3. In step S107, the final error (E_2_) is calculated in the non-invasive predicted value (Z) with respect to a reference value (Y). The goal is to train AdaBoost models for each cluster based on the least MARD and RMSE considering in-between patient variations. Multiple hyperparameters are changed to optimize each AdaBoost model to achieve these goals as shown in steps S108 and S109. This optimization process continues by going back to step 101 and S105 until the desired goal is achieved. Once the desired goals are achieved, we save the optimized hyperparameters of each model as shown in step S110. Step S111 is the end of building the AdaBoost models.

#### 3.2.2. Personalizing the Device for a Patient

Once AdaBoost models are successfully trained, then personalized calibration starts for a patient based on the individual patients’ characteristics such as food intake, physical activity, stress level, and skin temperature as shown in step 2 of Figure 3. Both non-invasive (X) and invasive (Y) values are measured for a patient, and paired data are collected, as shown in steps S201 and S202. The collected paired data is saved and initial error (E_1_) is calculated as shown in steps S203 and S204. Each AdaBoost model is recalled from step S110 and retrained for a specific patient in step S205. The retraining of the models is done based on the patient’s characteristics. Retrained models predict the non-invasive blood glucose values in step S206 and calculate the final error (E_2_) in step S207. Once again, hyperparameters are updated until the desired accuracy goals are achieved for a patient, as shown in steps S208 and S209. The software saves the models hyperparameters for a patient based on the least MARD and RMSE in step S210. Once the software starts predicting non-invasive value (Z) based on the updated models within the pre-defined accuracy limit, the calibration stage is completed, as shown in step S211.

#### 3.2.3. Patient Measure Blood Glucose Non-Invasively

Once the error in “non-invasive predicted (Z)” is within acceptable limit for the next several subsequent readings, the patient is notified to use the device non-invasively only to measure the blood glucose level, as shown in step S301 of Figure 3. At this point, blood glucose is measured non-invasively, and the device becomes personalized for a user.

#### 3.2.4. Periodic Re-Calibration of the Device (If Needed)

As part of the device periodic re-calibration, the patient measures both non-invasive (X) and invasive (Y) blood glucose for the next few cases, as shown in step S401 of Figure 3. The error (E_2_) in predicted non-invasive value (Z) is calculated in step S402. If error (E_2_) is within the acceptable limit, then the patient continues to use the device non-invasively, as shown in steps S403 and S404. If predicted non-invasive glucose value is outside the acceptable limit, then the device undergoes personalized calibration again, as demonstrated in step 2 of Figure 3.

### 3.3. PGMS Algorithm Development

The non-invasive glucose ($x_{i}$) is measured with respective invasive reference values ($y_{i}$). The relative difference (d_i_) in non-invasive measured value is calculated by Equation (11). For the AdaBoost model, $x_{i}$ is the independent variable, and $d_{i}$ is the dependent variable. Initially, data ($x_{i},\;d_{i}$) develops weak predictors ($h_{t}$) as shown in Equation (12).

(11) $$d_{t}=y_{i}-x_{i}$$

(12) $$h_{t}(x_{i})\to d_{i}$$

The error between predicted and the actual relative difference is calculated in terms of loss for each training data by Equation (13).

(13) $$l_{t}(i)=\left|h_{t}(x_{i})-d_{i}\right|$$

The average loss is calculated from Equation (7) based on linear, square, or exponential loss functions. The confidence in predictor is calculated in the same way as shown in Equation (8). The weight is updated by Equation (14). By using Equation (14), the probability is updated for each weak predictor.

(14) $$D_{t+1}(i)=D_{t}(i)\times \beta_{t}{}^{\left(\alpha \times (1-L_{t}(i))\right)}$$

In the end, strong predictor in terms of the error prediction model is derived by Equation (15) until it reaches the optimized result. For every non-invasive measured value ($x_{i})$, predicted relative difference ($d_{ipred}$) is calculated during the model training and optimization. Once predicted relative difference ($d_{ipred}$) is estimated, we calculate non-invasive predicted blood glucose value $z_{i}$ from Equation (16).

(15) $$d_{ipred}=\inf\left[d_{i}\in R:\sum_{t:h_{t}(x_{i})\leq d_{i}}\alpha \times \log\left(\frac{1}{\beta_{t}}\right)\geq \frac{1}{2}\alpha \times \sum_{t}\log\left(\frac{1}{\beta_{t}}\right)\right]$$

(16) $$z_{i}=x_{i}+d_{ipred}$$

The iteration continues until RMSE as calculated in Equation (17) is within an acceptable range and reached optimality to predict the relative difference in blood glucose values accurately.

(17) $$RMSE=\sqrt{\frac{1}{m}\sum_{i=1}^{m}{\left(d_{i}-d_{ipred}\right)}^{2}}$$

To ensure prediction is accurate, we calculate the initial MARD (E_1_) in the non-invasive measurement by Equation (18) and compare it with final MARD (E_2_) in the non-invasive predicted as calculated by Equation (19). We expect a reduction in the final MARD to prove our approach.

(18) $$E_{1}=\frac{\sum_{i=1}^{m}\left|\frac{(y_{i}-x_{i})\times 100}{y_{i}}\right|}{m}$$

(19) $$E_{2}=\frac{\sum_{i=1}^{m}\left|\frac{(y_{i}-z_{i})\times 100}{y_{i}}\right|}{m}$$

Here,
$x_{i}$ = Measured non-invasive blood glucose value;X = Dataset of $x_{i}$;$y_{i}$ = Reference invasive blood glucose value;Y = Dataset of $y_{i}$;$z_{i}$ = Non-invasive predicted blood glucose value;Z = Dataset of $z_{i}$;$d_{i}$ = Relative different in measured non-invasive;$h_{t}$ = Weak predictor at t^th^ iteration;$d_{ipred}$ = Predicted relative different;$l_{t}\left(i\right)$ = Loss of each training data at t^th^ iteration;i = i^th^ paired data, which varies from 1,2,….m;β_t_ = Measure of confidence at t^th^ iteration;m = Number of paired data;t = t^th^ iteration, which is varies from 1,2,….T;α = Learning rateRMSE = Root mean square errorE_1_ = MARD before applying AdaBoost;E_2_ = MARD after applying AdaBoost;

### 3.4. Software Implementation for PGMS

We develop our code in python language version 3.7 to implement the AdaBoost algorithm represented from Equation (11) to Equation (19). We have used the Scikit-learn library, which has a wide variety of machine learning algorithms [41]. The Scikit-learn AdaBoost regressor has multiple hyperparameters that need to be optimized. These hyperparameters are regressor type, maximum depth, number of estimators, learning rate, loss function, and number of random states [41]. The training to test ratio is set at 70:30. We used a bagging regressor to randomize the data for training and testing to remove the biasing (if any). We also used the Pandas library to develop the CEGA plot for the non-invasive measured values and predicted values with respect to reference invasive values.

The inputs in our software implementation are non-invasive blood glucose values (X) as an independent variable and invasive blood glucose values (Y) as a dependent variable. The outputs of our implementation are non-invasive predicted blood glucose values (Z), which are expected to be the same as reference invasive blood glucose values (Y). AdaBoost model is trained on 70% of data to predict non-invasive blood glucose values (Z) as a function of non-invasive values (X). Once the model is built, the test is conducted on the remaining 30% data to check the prediction efficacy. After each test run, code is written to compare the initial and final accuracies in terms of MARD to check the prediction accuracy using Equations (18) and (19). The RMSE is calculated by Equation (17) to test the robustness of prediction. Every run is followed by the CEGA plot with the calculation of the number of paired data in Zones A, B, C, D, and E. We compared the improvement in the CEGA plot Zone by Zone based on the initial paired data (X, Y) to final paired data (Z, Y). 

We changed various regressors named as decision tree, gradient boosting, and support vector machine during each run to optimize the test result. The maximum depth was changed from 1 to 100 and estimators were varied from 10 to 500. The learning rate was set with different values ranging from 0.0001 to 1 and random state was changed from 1 to 5. We chose different loss functions like linear, square, and exponential during the optimization run. We performed the grid search for model tuning and hyperparameter optimization until the desired MARD and CEGA plot in non-invasive predicted values was achieved.

### 3.5. Baseline Simulation Datasets

For the GlucoTrack non-invasive blood glucose monitor, a clinical study was conducted on 91 subjects consisting of type 1 and type 2 diabetes patients [18]. It had 1772 paired data varying from 65~492 mg/dl and 80~352 mg/dl from the HemoCue reference invasive and GlucoTrack non-invasive devices, respectively [18]. We produced the “dataset 1” statically, which consists of 918 paired invasive and non-invasive values similar to that of GlucoTrack and HemoCue [18]. The dataset 1 has the same invasive range (65~492 mg/dl), non-invasive range (80~352 mg/dl), and variation (−221%~61%) as listed in Table 5. The most important parameter MARD of dataset 1 is 23.9% compared to 23.4% of CoG data as shown in Table 5, which is within ±1% accuracy.

For the CoG non-invasive blood glucose monitor, a clinical study was conducted on 19 subjects consisting of type 1 and type 2 diabetes patients [19]. It had 730 paired data varying from 37~458 mg/dl and 40~428 mg/dl from the Okmeter Match reference invasive and CoG non-invasive devices, respectively [19]. We produced “dataset 2”, consisting of 470 paired reading similar to CoG and Okmeter. The simulated dataset 2 has the same invasive range (37~458 mg/dl), non-invasive range (40~428 mg/dl), and variation (−131%~65%) as listed in Table 6. The MARD for dataset 2 is 17.4% compared to 17.1% of CoG data as shown in Table 6, which is within ±1% accuracy. 

## 4. Results and Discussion

In this section, we present the result of the different approaches applied in the PGMS. This section also elaborates on the initial challenges faced and the resolution of those challenges.

### 4.1. Initial Approaches and Challenges for PGMS

#### 4.1.1. PGMS Using the AdaBoost without Clustering

Initially, we apply the AdaBoost algorithm without clustering using our PGMS software to the dataset 1. The model is trained using the 642 paired data (~70% of 918 paired data of the dataset 1). We tuned the AdaBoost model after several rounds of hyperparameters optimization using the grid search technique. During each run, the test is performed on the remaining 276 paired data (~30% of 918), randomly. The best value of the final MARD (E_2_) is achieved as 26.3% on the non-invasive predicted values compared to 27.4% as the initial MARD (E_1_) on non-invasive measured values as summarized in third and fourth columns of Table 7. A very slight improvement in the MARD is observed. In order to further improve the MARD, we apply the Clustering technique as discussed in the next section.

#### 4.1.2. PGMS with the K-Means Clustering and AdaBoost

The dataset 1 had a very wide range of blood glucose values, from 65 to 492 mg/dl for the invasive values and 80 to 352 mg/dl for the non-invasive measured values, as shown in Table 5. Hence, initial error (E_1_) had an extensive range of variations from −221 to 61%. Therefore, we divided the data into different groups using the K-means clustering algorithm. Then, we applied the AdaBoost algorithm on each cluster separately to predict the non-invasive blood glucose values. We trained and tested the models for each cluster with different cluster sizes like 2, 3, 4, 5, and 6. Unfortunately, there is a very slight improvement in accuracy for each case. Cluster with size as 4 has a better result and hence result is presented in fifth and sixth columns of Table 7. Overall MARD is calculated as the weighted average of the MARD of 4 clusters. We achieved final MARD (E_2_) as 25.1% in the non-invasive predicted values compared to 26.8% of initial MARD (E_1_). There is a very minor reduction in the error and improvement in the accuracy. We can easily interpret that K-means clustering combined with AdaBoost was also unable to predict the non-invasive blood glucose values accurately.

### 4.2. PGMS with Domain-Knowledge Clustering and AdaBoost

In order to further improve the accuracy, we have divided the entire paired data into different clusters based on our domain-knowledge. We formed the six clusters inspired by patient conditions and blood glucose values, as shown in Table 8. We named these clusters as hypoglycemia (< 80 mg/dl), non-diabetic (81–115 mg/dl), pre-diabetic (116–150 mg/dl), diabetic (151–180 mg/dl), highly diabetic (181–250 mg/dl), and critically diabetic (> 250 mg/dl). During the personalized calibration, based on the patient’s blood glucose range, some of the models among the trained AdaBoost models were selected for a patient. For example, if the blood glucose varies from 95~162 mg/dl for a patient throughout the day during the personalized calibration, the selected cluster and trained models are no diabetic range (81–115 mg/dl), pre-diabetic range (116–150 mg/dl), and diabetic range (151–180 mg/dl) to predict the non-invasive blood glucose value. Furthermore, based on the non-invasive measured blood glucose value, the final model will be shortlisted.

We divided the paired input data (X, Y) into six clusters as defined in Table 8. Each cluster is trained separately by the AdaBoost algorithm to predict accurate non-invasive blood glucose values (Z) using 70% paired data. Hyperparameters were optimized using the grid search for model tuning. Once optimization was over, the test was performed on the remaining 30% paired data to calculate the improvement in mean, minimum, and maximum relative difference in the non-invasive predicted values (Z) for each cluster. Overall initial MARD (E_1_) and final MARD (E_2_) are calculated as the weighted average of the mean relative difference of each cluster, as shown in Equations (20) and (21). The overall RMSE is calculated by Equation (22) to check the prediction robustness of the trained model.

(20) $$E_{1}=\frac{1}{n}\sum_{j=1}^{n}m_{j}\sum_{i=1}^{m_{j}}\frac{1}{m_{j}}\left|\frac{y_{ij}-x_{ij}}{y_{ij}}\right|\times 100$$

(21) $$E_{2}=\frac{1}{n}\sum_{j=1}^{n}m_{j}\sum_{i=1}^{m_{j}}\frac{1}{m_{j}}\left|\frac{y_{ij}-z_{ij}}{y_{ij}}\right|\times 100$$

(22) $$RMSE=\sqrt{\frac{1}{m}\sum_{j=1}^{n}m_{j}\times RMSE_{j}^{2}}$$

Here,
n = Number of clusters;j = Cluster number, which is from 1,2,….n;m_j_ = Number of paired data in the j^th^ cluster;i = i^th^ paired data, which varies from 1,2,….m;$x_{ij}$ = Measured non-invasive blood glucose value for i^th^ paired data in j^th^ cluster;$y_{ij}$ = Reference invasive blood glucose value for i^th^ paired data in j^th^ cluster;$z_{ij}$ = Non-invasive predicted blood glucose value for i^th^ paired data in j^th^ cluster;E_1_ = Overall MARD in percentage before applying the AdaBoost;E_2_ = Overall MARD after applying the AdaBoost;RMSE_j_ = Root mean square error of jth cluster

#### 4.2.1. Results of the PGMS on Dataset 1

For dataset 1, the AdaBoost model is trained with 642 paired data (~70% of 918) consisting of reference invasive and non-invasive measured values. During the model tuning, the best test result to predict the non-invasive blood glucose values with least MARD and best CEGA plot is achieved for the hyperparameters set as decision tree regressor, 10 maximum depths, 200 estimators, 0.7 learning rate, exponential loss function, and 3 random states as shown in the third column of Table 9. 

Later, the test is performed on a trained and optimized model with the remaining 276 paired test data (~30% of 918). The optimized test result is presented in Table 10. 

The first column of Table 10 is a cluster type. It consists of 6 clusters based on the different ranges of blood glucose values. For each cluster, we summarized the % minimum error, % maximum error, and % MARD calculated in the non-invasive measurements (X). Subsequently, the % minimum error, % maximum error, and % MARD were calculated for the non-invasive predicted (Z). The AdaBoost model predicts the non-invasive values accurately and hence reduces the final errors compared to the initial errors for each cluster as shown in Table 10. 

The last three rows of Table 10 summarize the overall result for the entire range (all clusters). The final MARD for the non-invasive predicted values is reduced to 7.3% compared to 25.4% of the initial MARD. The minimum error is reduced to −32% for the non-invasive predicted values compared to −152% in the non-invasive measured values. The maximum error is reduced to 22% for the non-invasive predicted values compared to 53% in the non-invasive measured values. The errors are drastically reduced by the successful prediction of non-invasive blood glucose values. The RMSE has reduced from 54.5 mg/dl to 20 mg/dl, which demonstrates the prediction robustness. Hence, it validates the concept of PGMS using domain-knowledge clustering and AdaBoost.

**Figure 4:**
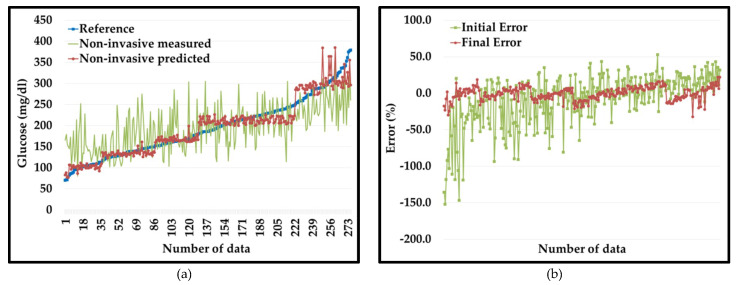
(**a**) Non-invasive measured (in green color) vs. non-invasive predicted (in red color) with respect to reference values (in blue color). (**b**) Percentage error non-invasive measured (in green color) vs. non-invasive predicted (in red color) for dataset 1.

We developed the plot of invasive (Y), non-invasive measured (X), and non-invasive predicted (Z) values for the dataset 1, shown in Figure 4a. It is very illustrative that initial values of non-invasive blood glucose (in green color) show extensive errors with respect to reference invasive blood glucose values (in blue color). However, non-invasive predicted values (in red color) curve follows the reference invasive blood glucose values curve (in blue color) as error reduces extensively.

Figure 4b shows a graphical representation of the initial errors (E_1_) in the non-invasive measured values (in green color) compared to the final errors (E_2_) in the PGMS (in red color). From the plot, it is very evident that initially non-invasive measured values have extensive errors ranging from −152%~53% (green color) compared to the errors in the non-invasive predicted values (red color) from −32~22% for the dataset 1.

**Figure 5:**
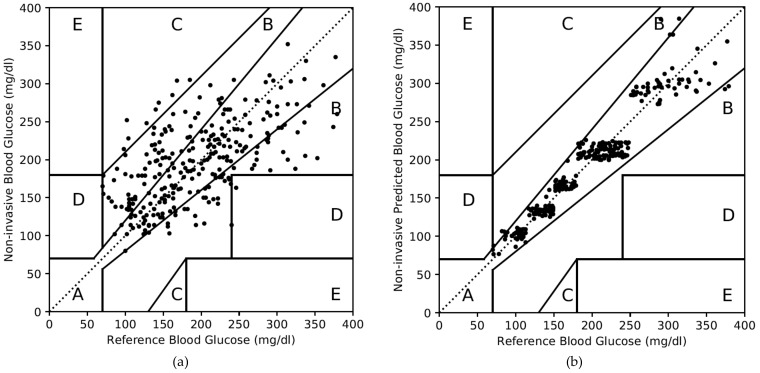
Clarke error grid analysis (CEGA) plot for the dataset 1. (**a**) For non-invasive measured values, (**b**) for non-invasive predicted values.

For dataset 1, the CEGA plot for the non-invasive measured values is shown in Figure 5a and summarized in the second and third columns of Table 11. Out of 276 test data, 149 and 115 test data fall in Zone A and B, respectively, as shown in the second column of Table 11. Zones C and D have 9 and 3 paired data, respectively. In terms of percentage, Zone A, B, C, and D consist of 54%, 42%, 3%, and 1%, respectively, as summarized in the third column of Table 11. Due to substantial error in the non-invasive measured value, only 54% of paired data is in Zone A of CEGA plot.

The CEGA plot for the non-invasive predicted values for dataset 1 is shown in Figure 5b. It is very illustrative that most of the paired data are part of Zone A; very few of Zone B; and none of Zones C, D, and E. The fourth and fifth columns of Table 11 summarize the CEGA plot as shown in Figure 5b. Zone A consists of 267 data (97%) and Zone B consists of 9 data (3%). Zones C, D, and E consist of none. This result is achieved due to the accurate prediction in the non-invasive blood glucose values by the PGMS concept based on the domain-knowledge clustering and AdaBoost algorithm.

#### 4.2.2. Results of the PGMS on Dataset 2

We also applied our approach to dataset 2. The AdaBoost model is trained with 329 paired data (~70% of 470) consisting of reference invasive and non-invasive measured values. The model is tuned with hyperparameters optimization using a grid search. The best result is achieved with the hyperparameters set as decision tree regressor, 20 maximum depths, 150 estimators, 0.008 learning rate, linear loss function, and 1 random state as shown in the fourth column of Table 9. The test is performed on a trained and optimized model with the remaining 143 paired test data (~30% of 470). The optimized test result is presented in Table 12.

For dataset 2, the AdaBoost model also predicts the non-invasive values accurately post domain-knowledge based clustering. It reduces the final errors compared to the initial errors for each cluster as shown in Table 12. The final MARD for the non-invasive predicted values is reduced to 7.1% compared to 18.4% of the initial MARD. The minimum error is reduced to −50% compared to −131% initially. The corresponding maximum error is reduced to 34% compared to 65% initially. The RMSE has reduced from 38.8 mg/dl to 13.9 mg/dl to show the prediction robustness. Once again, the errors are drastically reduced by the successful prediction of non-invasive blood glucose values. Hence, it re-validates the concept of PGMS with improved accuracy.

**Figure 6:**
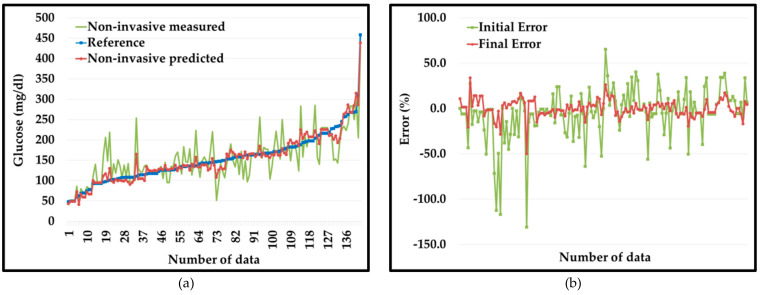
(**a**) Non-invasive measured (in green color) vs. non-invasive predicted (in red color) with respect to reference values (in blue color). (**b**) Percentage error non-invasive measured (in green color) vs. non-invasive predicted (in red color) for dataset 2.

We developed the plot of invasive (Y), non-invasive measured (X), and non-invasive predicted (Z) values for the dataset 2 and shown in Figure 6a. It is very illustrative that initial values of non-invasive blood glucose (in green color) show extensive deviation from reference invasive blood glucose values (in blue color). However, non-invasive predicted values (in red color) curve follows the reference invasive blood glucose values curve (in blue color) very well as error reduces drastically.

Figure 6b shows a graphical representation of the initial errors (in green color) compared to the final errors (in red color). From the plot, it is very evident that initially non-invasive measured values have very wide errors ranging from −131~65% (green color) compared to the errors in the non-invasive predicted values (red color) from −50~34% for the dataset 2.

**Figure 7:**
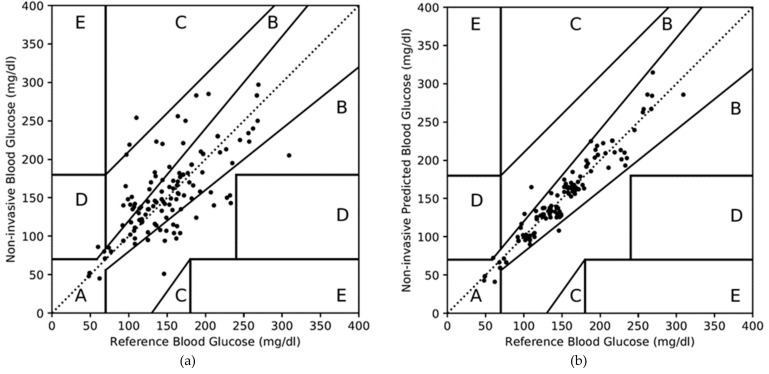
Clarke error grid analysis (CEGA) plot for the dataset 2. (**a**) For non-invasive measured values, (**b**) for non-invasive predicted values.

For dataset 2, the CEGA plot for the non-invasive measured values is shown in Figure 7a and summarized in the second and third columns of Table 13. Out of 143 test data, 99 and 41 test data fall in Zones A and B, respectively, as shown in the second column of Table 13. Zones C and D have 2 and 1 paired data, respectively. In terms of percentage, Zones A, B, C, and D consist of 69%, 29%, 1%, and 1%, respectively, as summarized in the third column of Table 13. Due to substantial error in the non-invasive measured value, only 69% of paired data is in the Zone A of CEGA plot.

The CEGA plot for the non-invasive predicted values for the dataset 2 is shown in Figure 7b. It is very illustrative that most of the paired data are part of Zone A; very few of Zone B; and none of Zones C, D, and E. The fourth and fifth columns of Table 13 summarize the CEGA plot as shown in Figure 7b. Zone A consists of 140 data (98%); Zone B consists of 3 data (2%); and there are none in Zones C, D, and E. Once again, this result is achieved due to the accurate prediction in the non-invasive blood glucose values by the PGMS.

We started with the AdaBoost alone to apply in the PGMS. However, the MARD was reduced from 27.4% to 26.3% for dataset 1 as summarized in Table 14. It improves accuracy very little due to large initial errors (−221% to 61%) in the non-invasive measured values. To resolve the large initial error issue, we divided the entire data into different groups using the K-means clustering technique and then applied AdaBoost for each cluster. However, the MARD was reduced from 26.8% to 25.1% for dataset 1 as listed in Table 14. It also did not produce a good result as K-means clustering forms the groups based on random centroid and density. Hence, we changed our clustering approach from the K-means algorithm to domain-knowledge.

We formed six different clusters based on the blood glucose range and patient diabetes condition. The AdaBoost algorithm started predicting the non-invasive blood glucose values accurately in each cluster. PGMS approach is applied to two different datasets and exceptional results are achieved. MARD was reduced from 25.4% to 7.3% for dataset 1 as shown in Table 14. The CEGA plots showed that 97% and 3% of data fall in Zones A and B after applying our approach compared to 54%, 42%, 3%, and 1% of data in Zones A, B, C, and D initially for the dataset 1. The minimum error was reduced from −152% to −32% and maximum error was reduced from 53% to 22%. To validate further, we applied our approach to dataset 2. The result echoed for dataset 2 and MARD was reduced from 18.4% to 7.1% as tabulated in Table 14. The CEGA plots showed that 98% and 2% of data falls in Zones A and B after applying our approach compared to 69%, 29%, 1%, 1% of data in Zones A, B, C, and D initially for the dataset 2. The minimum error was reduced from −131% to −50% and maximum error was reduced from 65% to 34%.

PGMS produces the industry’s best result for non-invasive blood glucose measurement compared to existing monitors as presented in Table 14. PGMS achieved the MARD as 7.1% compared to 23.4% of GlucoTrack, 17.1% of CoG, 13.8% of SugarBEAT, 12.3% of Symphony, and 14.8% of Eversense. Wizmi achieved the MARD at 7.2%, which is very close to PGMS MARD. However, on the CEGA plot, PGMS has achieved 98% of paired data in Zone A and 2% in Zone B compared to 93% and 7%, respectively, for Wizmi.

## 5. Conclusions and Future Work

In this study, we presented a novel data-driven machine learning based personalized glucose monitoring system (PGMS) for non-invasive blood glucose measurement. PGMS approach used the domain-knowledge clustering technique and AdaBoost algorithm to train the error prediction models and predicted the non-invasive blood glucose values accurately. We validated the PGMS concept by applying it to two different datasets. The PGMS achieved the final MARD as 7.3% on dataset 1 and 7.1% on dataset 2 for non-invasive predicted values. Moreover, the CEGA plots on dataset 1 showed that 97% of predicted non-invasive values fall in Zone A and 3% lie in Zone B after applying the PGMS concept. Similarly, the CEGA plots on dataset 2 showed that 98% of non-invasive predicted values lie in Zone A and 2% in Zone B. For both datasets, there are no non-invasive predicted values under the Zones C, D, and E. The extraordinary result of PGMS is a crucial step towards an accurate non-invasive blood glucose measurement for diabetes management. In a future study, we also aim to perform clinical trials to improve the result by collecting personalized data such as food intake, physical activity, stress level, and skin temperature to enhance the accuracy of PGMS. 

## 6. Patents

Research presented in this paper has filed for a patent (not published yet) in the Korea and USA patent offices with application number SKP19-0018KR and docket number 16/777,033, respectively. The title of the invention is “PERSONALIZED NON-INVASIVE BLOOD GLUCOSE MEASUREMENT DEVICE AND METHOD USING THE MEASUREMENT DEVICE.”

## Figures and Tables

**Table 1:** Regulations for accurate blood glucose measurement.

Regulation	Glucose Level	Acceptable Error Range	Device Accuracy	MARD
US Food and Drug Administration (FDA) [24,25]	Entire range	−15%~15%	> 95%	< 5%
		−20%~20%	> 99%	< 1%
European Medicines [26] Health Canada [27] China Food and Drug Administration (CFDA) [28]	≥100 mg/dL	−15%~15%	> 95%	< 5%
	<100 mg/dL	−15~15 mg/dL	> 95%	< 5%

**Table 2:** FDA approved invasive blood glucose monitors with accuracies.

Product Name	Manufacturer	Accuracy
Contour Next [9]	Bayer	100%
Accu-Check Aviva Plus [9]	Roche	99%
Walmart ReliOn Confirm [9]	Arkray	97%
CVS Advanced [9]	AgaMatrix	96%
FreeStyle Lite [9]	Abbott Diabetes Care	96%

**Table 3:** Comparison of non-invasive sensor technologies with their accuracies.

Sensing Technology	MARD (%)	CEGA Plot (%)		
		Zone A	Zone A and B	Zone C, D, and E
Infrared spectroscopy [10]	-	84.0	-	-
Impedance spectroscopy [11]	-	56.0	93.0	7.0
Diffuse reflectance [12]	-	87.5	95.8	4.2
Raman spectroscopy [13]	-	86.7	-	-
Optical coherence tomography [14]	11.5	83.0	99.0	1.0
Photo-acoustic spectroscopy [15]	11.8	82.7	100.0	0.0
Multi-sensor^1^ [16]	8.3	90.0	100.0	0.0
Multi-sensor^2^ [17]	8.8	92.7	100.0	0.0
Multi sensor^3^ [18]	22.4	60.0	96.0	4.0

Multi-sensor^1^ consists of near-infrared and impedance spectroscopy. Multi-sensor^2^ consists of near-infrared and photo-acoustic spectroscopy. Multi sensor^3^ consists of thermal, electromagnetic, ultrasonic.

**Table 4:** Non-invasive and minimally-invasive blood glucose monitors with their accuracies.

Product Name	Manufacturer	Sensing Technology	Accuracy
GlucoTrack [18]	Integrity Application	Multi-technology (Ultrasound + Thermal + Electromagnetic)	MARD: 23.4% Zone A: 57% Zone B: 39%
Combo Glucometer (CoG) [19]	CNOGA Medical	Near-Infrared Spectroscopy	MARD: 17.1% Zone A: 86.2% Zone B: 12.6%
SugerBEAT [20]	Nemaura Medical	Reverse Iontophoresis	MARD: 13.8%
Symphony [21]	Echo Therapeutics	Sonophoresis	MARD: 12.3% Zone A: 81.7% Zone B: 18.3%
Wizmi [22]	Wear2b Ltd.	NIR spectroscopy	MARD: 7.2% Zone A: 93% Zone B: 7%
Eversense [23]	Senseonics	Fluorescence	MARD: 14.8%

**Table 5:** Baseline dataset 1.

Parameters	Unit	GlucoTrack [18]	Dataset 1
Invasive Range	mg/dl	65~492	65~492
Non-invasive range	mg/dl	80~352	80~352
Number of paired data	-	1772	918
MARD	%	23.4	23.9
Minimum Error	%	−221	−221
Maximum Error	%	61	61

**Table 6:** Baseline dataset 2.

Parameters	Unit	CoG [19]	Dataset 2
Invasive Range	mg/dl	37~458	37~458
Non-invasive range	mg/dl	40~428	40~428
Number of paired data	-	730	470
Minimum Error	%	−131	−131
Maximum Error	%	65	65
MARD	%	17.1	17.4

**Table 7:** Error and MARD reduction by different initial approaches.

Parameter	Unit	AdaBoost		AdaBoost + K-Means Clustering	
		Initial Error	Final Error	Initial Error	Final Error
Minimum Error	%	−60.1	−60.7	−61	−57
Maximum Error	%	149.3	143.5	149	139
MARD	%	27.4	26.3	26.8	25.1

**Table 8:** Clusters formed based on domain-knowledge.

Blood Glucose Range (mg/dL)	Cluster Name
50–80	Hypoglycemia
81–115	No diabetic
116–150	Pre-diabetic
151–180	Diabetic
181–250	Highly diabetic
> 250	Critically diabetic

**Table 9:** Optimized hyperparameters values.

Hyperparameters	Ranges	For Dataset 1	For Dataset 2
Regressor type	Decision Tree Gradient Boosting Support Vector Machine	Decision Tree	Decision Tree
Depth	1~100	10	20
Estimators	10~500	200	150
Learning rate	0.0001~1	0.7	0.008
Loss function	Linear, Square Exponential	Exponential	Linear
Random state	1~5	3	1

**Table 10:** Results of the PGMS applied to dataset 1.

Cluster	Paired Data	Parameter	Unit	Initial Error	Final Error
<80	4	Minimum	%	−152	−23
		Maximum	%	−92	2
		MARD	%	124.6	12.6
81–115	32	Minimum	%	−147	−30
		Maximum	%	20	19
		MARD	%	48.3	7.9
116–150	51	Minimum	%	−94	−16
		Maximum	%	21	13
		MARD	%	31.2	6.0
151–180	42	Minimum	%	−81.0	−18.3
		Maximum	%	34.8	7.1
		MARD	%	21.8	5.1
181–250	93	Minimum	%	−64.9	−21.5
		Maximum	%	52.5	16.3
		MARD	%	16.6	7.5
>250	54	Minimum	%	−12	−32
		Maximum	%	43	22
		MARD	%	17.2	9.0
Total	276	Minimum *	%	−152	−32
		Maximum †	%	53	22
		Overall MARD ‡	%	25.4	7.3

* Minimum is lowest out of 6 clusters. † Maximum is the highest out of 6 clusters. ‡ Overall MARD is the weighted MARD of 6 clusters.

**Table 11:** CEGA plot summary for the dataset 1.

Zones	Non-Invasive Measured Values		Non-Invasive Predicted Values	
	Number	%	Number	%
Zone A	149	54	267	97
Zone B	115	42	9	3
Zone C	9	3	0	0
Zone D	3	1	0	0
Zone E	0	0	0	0

**Table 12:** Results of the PGMS applied to the dataset 2.

Cluster	Paired Data	Parameter	Unit	Initial Error	Final Error
<80	12	Minimum	%	−43.3	−20.6
		Maximum	%	27.4	33.8
		MARD	%	11.3	10.9
81–115	26	Minimum	%	−130.9	−49.8
		Maximum	%	16.7	16.6
		MARD	%	31.7	9.8
116–150	39	Minimum	%	−64.0	−15.4
		Maximum	%	65.1	26.0
		MARD	%	17.0	6.5
151–180	30	Minimum	%	−56.1	−14.2
		Maximum	%	40.1	8.8
		MARD	%	15.6	4.5
181–250	27	Minimum	%	−50.5	−19.6
		Maximum	%	38.6	17.1
		MARD	%	16.5	7.3
>250	9	Minimum	%	−10.4	−17.0
		Maximum	%	33.7	7.5
		MARD	%	11.1	5.6
Total	143	Minimum *	%	−131	−50
		Maximum †	%	65	34
		Overall MARD ‡	%	18.4	7.1

* Minimum is the lowest out of 6 clusters. † Maximum is the highest out of 6 clusters. ‡ Overall MARD is the weighted MARD of 6 clusters.

**Table 13:** CEGA plot summary for dataset 2.

Zones	Non-Invasive Measured Values		Non-Invasive Predicted Values	
	Number	%	Number	%
Zone A	99	69	140	98
Zone B	41	29	3	2
Zone C	2	1	0	0
Zone D	1	1	0	0
Zone E	0	0	0	0

**Table 14:** Performance comparison of PGMS with other non-invasive or minimal-invasive monitors.

Non-Invasive Measurement System		MARD	CEGA Plot	
			Zone A	Zone B
PGMS	AdaBoost	26.3%	-	-
	K-Means Clustering + AdaBoost	25.1%	-	-
	Domain-knowledge Clustering + AdaBoost	7.1%	98%	2%
GlucoTrack		23.4%	57%	39%
CoG		17.1%	86.2%	12.6%
SugarBEAT		13.8%	-	-
Symphony		12.3%	81.7%	18.3%
Wizmi		7.2%	93%	7%
Eversense		14.8%	-	-
